# Protein Dynamics in Cytosolic DNA-Sensing Antiviral Innate Immune Signaling Pathways

**DOI:** 10.3389/fimmu.2020.01255

**Published:** 2020-07-02

**Authors:** Chunfu Zheng

**Affiliations:** ^1^Department of Immunology, School of Basic Medical Sciences, Fujian Medical University, Fuzhou, China; ^2^Department of Microbiology, Immunology and Infectious Diseases, University of Calgary, Calgary, AB, Canada

**Keywords:** protein dynamics, protein trafficking, conformational change, DNA sensing signaling, innate immunity

## Abstract

Antiviral innate immunity works as the first line of host defense against viral infection. Pattern recognition receptors (PRRs) and adaptor proteins involved in the innate immune signaling pathways play critical roles in controlling viral infections via the induction of type I interferon and its downstream interferon-stimulated genes. Dynamic changes of adaptor proteins contribute to precise regulation of the activation and shut-off of signaling transduction, though numerous complex processes are involved in achieving dynamic changes to various proteins of the host and viruses. In this review, we will summarize recent progress on the trafficking patterns and conformational transitions of the adaptors that are involved in the antiviral innate immune signaling pathway during viral DNA sensing. Moreover, we aim to dissect the relationships between protein dynamics and DNA-sensing antiviral innate immune responses, which will reveal the underlying mechanisms controlling protein activity and maintaining cell homeostasis. By comprehensively revealing protein dynamics in cytosolic DNA-sensing antiviral innate immune signaling pathways, we will be able to identify potential new targets for the therapies of certain autoimmune diseases.

## Introduction

Cellular processes are dependent on transmembrane receptors to communicate and transmit various signals into intracellular compartments. Dynamic changes in proteins precisely regulate the activation and inhibition of the signaling transduction. As the host's frontline defense against viral infection, antiviral innate immunity is mainly triggered by the interaction between pattern recognition receptors (PRRs) and viral pathogen-associated molecular patterns (PAMPs), followed by the activation of downstream adaptor proteins, such as stimulator of interferon genes (STING), mitochondrial antiviral signaling protein (MAVS), tumor necrosis factor receptor-associated factor (TRAFs), Toll/IL-1 receptor domain-containing adaptor inducing IFN-β (TRIF), and some other adaptors, which contribute to the induction of type I interferons (IFN-I). Therefore, the expression of IFN-stimulated genes (ISGs) is significantly increased to restrict viral infection or replication (1).

In host antiviral innate immunity signaling pathways, protein trafficking is one of the primary protein dynamics essential for the activation of the signaling pathway. Until 2013, the cyclic GMP-AMP synthase (cGAS) had been identified as the only universal cytoplasmic DNA sensor in various cell types to sense double-stranded DNA (dsDNA) and catalyze the synthesis of cyclic GMP-AMP (cGAMP) (2). STING, the only receptor of cGAMP, moves from the endoplasmic reticulum (ER) to the Golgi and recruits TANK binding kinase 1 (TBK1) and interferon regulatory factor 3 (IRF3). The location of TBK1 and IRF3 on Golgi engages their interaction with each other and phosphorylation transition. Activated IRF3 in the nucleus directly binds to the promoter region of IFN-I to enhance the transcription of IFN-β (3, 4). Toll-like receptors (TLRs) also play critical roles in antiviral innate immunity. TLR3, TLR7, and TLR9 are the first subgroup of PRRs identified in mammals. They traverse from ER to endosome by the transmembrane protein UNC93B1 to recognize viral PAMPs and induce innate immunity (5). The mitochondrially located protein MAVS transmits downstream signaling of antiviral innate immunity, with signaling complexes assembling on the mitochondrial-associated ER membrane (MAM) (6). Thus, protein trafficking and different sub-cellular localizations contribute significantly to the innate immune signal transduction.

The conformational transition is another type of protein dynamic for activation and signaling transduction. When sensing cytosolic dsDNA, cGAS needs to form into a polymer to bind to the dsDNA directly. Polymerization of cGAS is necessary for its enzymatic activity to catalyze the synthesis of cGAMP from ATP and GTP (7, 8). cGAMP works as the endogenous second messenger and binds to STING. Upon binding with cGAMP, STING undergoes the formation of the dimers and higher-order oligomers. A closed conformation of STING is formed following a 180° “twisting” of the STING dimer on its transmembrane domain upon ligand binding, leading to the oligomerization of STING through side-by-side packing of dimeric STING molecules, which is essential for STING trafficking and TBK1 trans-autophosphorylation (9, 10). Evidence indicates that homodimerization or heterodimerization of some TLRs in endosomes is critical for TLR-sensing PAMP (11). Besides, a recent study pointed out that the cleavage and release of the UNC93B-TLR9 complex in endosomes are required for activation of the signaling pathway (12). The retinoic acid-inducible gene I (RIG-I) is proposed to expose the N-terminal pair of caspase activation recruitment domains (CARDs), enabling an interaction with MAVS and thereby initiating downstream signaling (13). Thus, protein kinetics, including protein trafficking and conformational transition, play critical roles in the activation of innate immune signaling pathways. Revealing the working pattern of adaptors, especially how adaptors coordinate with other proteins in sub-cellular localization changing and conformational conversion in cytosolic DNA-sensing signaling pathways, is a matter of vital importance in innate immune responses.

## Protein Trafficking Patterns in cGAS-Sting Mediating IFN-β Production

DNA-sensing signaling is one of the main pathways in response to DNA virus infection that prevents viral invasion and replication intracellularly. Activation of DNA sensors and the adaptors directly regulates IFN-I production (14). Trafficking or different localizations of DNA sensors and their downstream adaptor proteins play essential roles in signal pathway activation. In the cGAS-STING signaling pathway, ER-retaining protein STING is usually thought to be activated by cGAMP (4). After its activation, STING traffics through the ER–Golgi intermediate compartment (ERGIC) and the Golgi apparatus in a process that is dependent on the cytoplasmic coat protein complex II (COPII) and ADP–ribosylation factor (ARF) GTPases (15). Located on Golgi, the kinase TBK1 and IRF3 are recruited by STING. A phosphorylation cascade allows signal transmission, leading to the activation of IRF3 and nuclear factor kappaB (NF-κB), which translocate into the nucleus to drive transcription of IFN-I and pro-inflammatory cytokines (16–18).

### Host Proteins Regulate the Trafficking of Adaptors for cGAS-STING Signal Transduction

Mukai et al. demonstrated that the palmitoylation of STING undergone on the Golgi was necessary for STING activation (19, 20). Also, STING trafficking to the Golgi is the pre-requisite for TBK1 and IRF3 recruitment, which is followed by phosphorylation and signal transduction to induce IFN-I production (21). Inactive rhomboid protein 2 (iRhom 2) and translocon-associated protein (TRAPβ) form a complex with STING and facilitate the STING trafficking from ER to Golgi (22, 23). Mitochondrial E3 ubiquitin-protein ligase 1 (MUL1), autocrine motility factor receptor (AMFR), and insulin-induced gene 1 (INSIG1) were also reported to play positive regulatory roles in promoting STING translocation to Golgi and activation through polyubiquitination modification on STING (24, 25). Gui et al. found that secretion-associated and RAS-related protein (SAR1A) and the COPII cargo-binding protein SEC24C could interact with STING and accelerate STING's trafficking to Golgi (26). After activation on Golgi, STING moves to perinuclear or other organelles. Gonugunta et al. confirmed that STING quickly moved to Rab7-positive endo-lysosomes after activation on Golgi for degradation, turning off the downstream signaling (27), while it has also been shown that sentrin-specific protease (SENP2), a putative regulator, promotes STING degradation in autophagosome (28). A recent study demonstrated that post-Golgi trafficking of STING regulated the autophagy signaling (29). Autophagy related gene 9a (Atg9a) controls dsDNA-driven dynamic translocation of STING and the innate immune responses (30). The transcription factor IRF3 is exported from the nucleus via the CRM1-mediated pathway to locate in the cytosol at rest stage, while it is imported into the nucleus when receiving the signal upstream (31). Phosphorylated IRF3 forms dimers and shuttles into the nucleus, where they interact with the coactivator CBP/p300 and initiate transcription of IFN-I and inflammatory cytokines (32, 33). Fas-associated factor 1 (FAF1) and DEAD BOX Helicase 56 (DDX56) are found to physically associate with IRF3-IPO5/importin-β3 complex, and overexpression of FAF1 or DDX56 reduces the interaction between IRF3 and IPO5/importin-β3 and disrupts the nuclear translocation of IRF3 (34, 35). Interestingly, a recent study showed that the adaptor protein TRIF, mainly involved in TLR signaling, also participates in cGAS-STING-mediated antiviral responses in a cell type-dependent manner (36).

### Viral Proteins Interfere With Adaptors Trafficking During cGAS-STING Signaling

Trafficking of STING and IRF3 nuclear importation are essential for the activation of the cGAS-STING signaling pathway. Various host proteins interact with STING or IRF3 to regulate their translocation and signaling transduction and to maintain homeostasis. Viruses, especially DNA viruses, have evolved multiple strategies to impede the cGAS-STING signaling pathway by dampening the trafficking of STING and IRF3. Herpes simplex virus type I (HSV-1) serine protease VP24 and serine/threonine kinase US3 were shown to target IRF3 and block its dimerization and nuclear translocation, with a subsequent reduction of IFN-I (37, 38). HSV-1 tegument proteins UL24 and UL42 were found to bind to the endogenous NF-κB subunits p65 and p50, abrogating their nuclear translocation and NF-κB activation downstream of the cGAS-STING signaling pathway during viral infection (39, 40).

**Figure 1:**
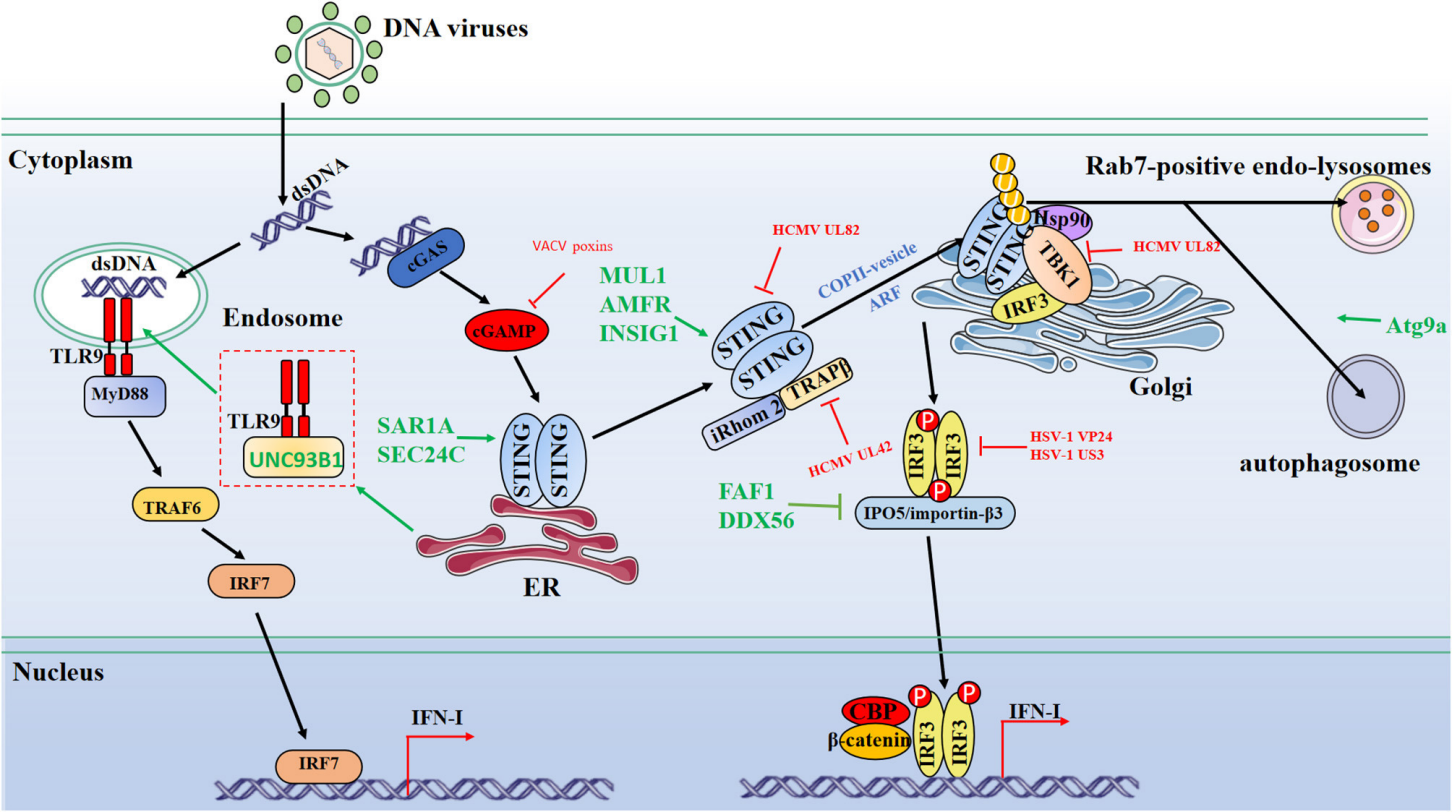
Schematic diagram of protein dynamics of cytosolic DNA-sensing signaling antiviral innate immunity signaling pathways. Cytosolic DNA sensors, such as cGAS and TLR9, recognize dsDNA and trigger IFN-I production through the transmission of a series of signals. Multiple steps in the DNA-sensing signaling pathways can be modulated by host and viral proteins. Green lines indicate that host proteins target adaptors. Red lines indicate that viral proteins interfere with adaptors. CBP, CREB-binding protein; P, phosphate; U, ubiquitin.

The Tegument protein UL82 of human cytomegalovirus (HCMV), one of the beta herpesviruses, was reported to impair STING-mediated signaling via two mechanisms. On the one hand, UL82 disrupts the STING-iRhom2-TRAPβ translocon complex and impedes the translocation of STING to Golgi. On the other hand, UL82 impairs the recruitment of TBK1 and IRF3 to the STING complex on Golgi, reducing IFN-I production (41). In addition, HCMV UL42 inhibits the trafficking and activation of STING by facilitating p62/LC3B-mediated degradation of TRAPβ (42). Recently, a study showed that vaccinia virus, a DNA virus replicating in the cytoplasm, encodes poxvirus immune nucleases (poxins) to restrict cGAS-STING signaling through cleaving 2′,3′- cGAMP and that deletion of poxin gene attenuates viral replication (43) (Figure 1).

## Translocation of TLRs Affects the DNA-Sensing Signaling

TLRs are one of the main subgroups of PRRs for the primary sensing of virus-derived nucleic acids, leading to the production of IFN-I, pro-inflammatory cytokines, and chemokines by the host cells (44). Evidence indicates that the transduction of the TLR signaling pathway is mainly dependent on its intracellular trafficking. To date, UNC93B1 is the unique trafficking vector identified for TLR3, TLR7, and TLR9. The interaction between UNC93B1 and TLRs facilitates its loading into COPII vesicles and transport through ERGIC to endosome, resulting in the production of IFN-I (45–47). To date, TLR9 is the only known DNA sensor among human TLRs (48). TLR9 is mainly expressed in endosomes among B cells, macrophages, and dendritic cells (DCs) (49). TLR9 recognizes unmethylated 2′-deoxyribo cytidine-phosphate-guanosine (CpG) DNA, which is mostly expressed in bacteria (50). It has been demonstrated that infection by certain DNA viruses activates the TLR9 signaling pathway, in which TLR9 interacts with myeloid differentiation factor 88 (MyD88). Subsequently, TLR9 forms a multiprotein signaling complex with Interleukin-1 Receptor-Associated Kinase 4 (IRAK4), IRAK1, TNF Receptor-Associated Factor 6 (TRAF6), TRAF3, and IkB kinase α and activates IRF7, which induces the production of IFN-I (51). An FYVE (Phe-Tyr-Val-Glu) finger-containing phosphoinositide (PI) kinase, PIKfyve, appears to play an important role in TLR9 trafficking and signal transduction in DCs and macrophages (52). Adaptor protein-3 (AP-3) was required for late-endosome localization of TLR9 to induce the production of IFN-I (53). In sum, the DNA receptor TLR9 cooperates with other cellular proteins to accurately control DNA-sensing signaling.

## Protein Conformational Transition Mediates the Signaling Activation

cGAS is one of the critical receptors that account for DNA-driven innate immune responses. The nucleotidyltransferase (NTase) domain, which consists of a central catalytic pocket and two separate surfaces with positive charges in the C-terminal of cGAS, is critical for the dimer formation and enzymatic activity (54). Upon binding to dsDNA, cGAS assembles into a cGAS-dsDNA oligomeric complex with two molecules of dsDNA embedded in two cGAS molecules (55, 56). cGAS dimers form ladder-like networks between two separate stretches of dsDNA or on one long crooked dsDNA helix, which markedly enhances the stability of each individual cGAS-dsDNA complex along the dsDNA (8, 57). In addition, subcellular fractionation and bio-chemical analysis suggest that cGAS is predominantly located on the plasma membrane through the N-terminal unstructured domain but not in the cytosol at rest stage. After DNA transfection, cGAS translocates to the cytoplasm and forms large foci (probably liquid droplets of cGAS-DNA complex), to respond to extraneous DNA and viral infection (58, 59).

As the only receptor of the second messenger cGAMP, CDN-binding domain (CBD) of dimeric STING binds asymmetric 2′,3′ cGAMP preferentially and is essential for the translocation of STING from ER to Golgi (60). STING polymer formation is necessary for recruiting TBK1. Phosphorylation transition during STING-TBK1-IRF3 complex formation requires a 180° rotation of the ligand-binding domain in STING since the binding site of STING-TBK1 is far away from the kinase active center of TBK1 (10). cGAMP induces the closing of the human STING homodimer and release of the STING C-terminal tail, which exposes a polymerization interface on the STING dimer and leads to the formation of disulfide-linked polymers via cysteine residue 148 (61). The hyperactive STING mutation typically results in serious autoimmune diseases by its constitutive release of C-terminal tail and polymerization (61).

TLRs have been notoriously difficult to crystallize, while more and more evidence shows that the conformational change of TLR plays an essential role in the binding of TLRs to its natural ligands. Upon addition of ligand, both TLR3 and TLR9 form dimers. However, full-length TLR9 in cells is suggested to exist as a pre-formed homodimer, and ligand binding simply induces a conformational change that is necessary for receptor activation (62).

## Concluding Remarks and Future Perspectives

Evidence has shown that protein trafficking in innate immunity is critical to signaling transduction. In DNA-sensing signaling pathways, the cGAS-STING pathway plays the main role in response to cytosolic DNA and antiviral innate immunity. The activation of ER-retained STING requires translocation from the ER to ERGIC and then to the Golgi. During translocation, STING activates IRF3 and NF-κB transcription factors that induce the expression of IFN-I and inflammatory cytokines. The STING signaling cascade is reported to be regulated by multiple checks, which are involved in different stages of STING activation and inhibition. Many studies have demonstrated that COPII-dependent vesicles, together with some other transmembrane protein complexes, play critical roles in STING trafficking and conformational transition in the activation and signaling transduction of STING (23, 63–65). Also, studies have shown that the degradation of activated STING, which plays an important role in maintaining cell homeostasis, is mediated by the ubiquitin-proteasome, lysosomal, or autophagic degradation pathway (27). It remains elusive which mechanism contributes most to the degradation of activated STING. Studies on the stringent trafficking patterns of STING post-Golgi will be crucial for revealing the underlying mechanism of homeostatic regulation of STING protein after activation. Moreover, it will provide a potential way to cure the autoimmune diseases caused by aberrant activation of STING.

Working as another DNA-sensing PRR, TLR9, translocated into endosome and released from UNC93B-TLR9 complex, senses CpG dsDNA, which is leaked from mitochondria, induces IFN-I production, and subsequently moves to the lysosome for degradation (66) (Figure 1). In addition, cellular proteins involved in different signaling pathways work together with TLR9 and regulate the translocation or conformational conversion, leading to various signal transduction and innate immunity responses. TLR9 is the only known TLR that recognizes CpG dsDNA, while other TLR members like TLR2, TLR3, and TLR4 show the capability to respond to the infection of DNA viruses, even though they do not sense viral dsDNA (67–69). Previous reports showed that hetero-dimerization and homo-dimerization of certain TLRs are essential for ligand binding and signaling transduction. One possibility is that these TLRs might recognize the viral dsDNA when they form hetero- or homo-dimers. It will be interesting to reveal how other TLRs function in DNA-sensing signaling, which will help our further understanding of how distinct TLR signal pathways are balanced.

## Author Contributions

CZ wrote, reviewed, and modified the manuscript.

## Conflict of Interest

The author declares that the research was conducted in the absence of any commercial or financial relationships that could be construed as a potential conflict of interest.
